# A digital intervention using virtual reality helmets to reduce dental anxiety of children under local anesthesia and primary teeth extraction: A randomized clinical trial

**DOI:** 10.1002/brb3.2600

**Published:** 2022-04-29

**Authors:** Qin Du, Xinru Ma, Shasha Wang, Shiyu Zhou, Chunmei Luo, Kun Tian, Wei Fei, Xianghong Liu

**Affiliations:** ^1^ Department of Stomatology Sichuan Academy of Medical Science & Sichuan People's Hospital Sichuan China; ^2^ School of Medicine University of Electronic Science and Technology Sichuan China; ^3^ School of Stomatology Zunyi Medical University Guizhou China; ^4^ Department of Stomatology North Sichuan Medical College Sichuan China; ^5^ Department of Stomatology Eastern Hospital Sichuan Academy of Medical Sciences & Sichuan Provincial People's Hospital Sichuan China

**Keywords:** child behavior, dental anxiety, distraction systems, pain perception, virtual reality exposure therapy

## Abstract

**Introduction:**

Behavior management of children during dental treatment is an important but challenging issue. As a new technique, VR has been applied in pediatric dental anxiety. But there is no final conclusion whether VR reduces children's dental anxiety.

**Methods:**

The aim of the study is to assess the effectiveness of a digital intervention using virtual reality (VR) helmets on dental anxiety, pain perception, and behavior triggered for children, as well as occurrence of simulator sickness in local anesthesia and primary teeth extraction. A total of 128 children, who needed primary teeth extraction under local anesthesia, were randomly allocated into two groups: use VR helmets and traditional behavior guidance procedures (control). Modified Child Fear Survey Schedule Dental Subscale (CFSS‐DS), Wong‐Baker FACES Pain Scale, Houpt Scale, and Simulator sickness questionnaire (SSQ) were used to assess children's dental anxiety, pain perception, and behavior triggered and occurrence of simulator sickness.

**Results:**

CFSS‐DS score in the VR group was significantly decreased after dental treatment (34.58±6.90 before operation and 32.32±15.58 after operation, *p *= .02). The score of Wong Baker Scale in the VR group (3.47±0.76) was significantly lower than that in the control group (5.56±1.13, *p *= .015). There was no significant difference in the Houpt Behavior Scale score and the SSQ score between the VR group and the control group (*p *= .35, *p *= .305).

**Conclusion:**

The use of VR helmets in primary teeth extraction can significantly reduce dental anxiety and pain perception in children without occurrence of simulator sickness.

## Graphical abstract

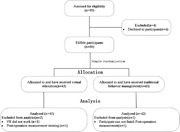

## 1 INTRODUCTION

Behavior management of children during dental treatment is an important but challenging issue. Dental department or dental treatment can trigger dental anxiety in children, which may increase pain perception and result in noncooperative behaviors and treatment avoidance (Seligman et al., 2017). Moreover, poorly managed pain impacts a child's well‐being and can lead to a reduced ability to cope effectively with future pain (Gates et al., 2020; Racine et al., 2016).

There are lot of behavior management tools, such as distraction, positive reinforcement, tell–show–do, and so on. Distraction is a widely used nonpharmacological technique for managing children during dental care. We use distraction to decrease the perception of unpleasantness and avert negative or avoidance behavior (Behavior Guidance for the Pediatric Dental Patient, 2018). There are various forms of distractors, including nondigital distractors and digital distractors, such as virtual reality (VR), television, tablets, smartphones, and so on (Gates et al., 2020).

VR has been defined as a “relatively new tool of human–computer interactions for a human becoming an active participant in a virtual world” (Gershon et al., 2004). It can be realized through several tools, including personal computer screens, mobile devices, and dedicated VR rooms. The most often used method in dental care is VR glasses or a head‐mounted helmet, which can be connected to a personal computer or linked to a mobile phone (Iannicelli et al., 2019). In recent years, VR has been applied in pediatric dental anxiety. There are several articles talking about it. It is a common view that VR can reduce children's pain perception during dental care (Chad et al., 2018; Kennedy et al., 1993; Niharika et al., 2018). But there is no final conclusion whether VR reduces children's dental anxiety (Custódio et al., 2020; Lahti et al., 2020; López‐Valverde et al., 2020; Niharika et al., 2018). Besides, the adverse events of VR applied in dental care, which seemed to be limited to mild simulator sickness, were not investigated before (Gates et al., 2020).

In this study, we cooperated with the manufacturer to develop a medical software used in VR system, which can interact with children to draw their attention and help them relax in dental treatment. The purpose of this randomized controlled clinical trial was to estimate the effectiveness of the digital intervention using VR helmets as a behavior management tool for children under local anesthesia and primary teeth extraction.

## 2 EXPERIMENTAL SECTION

This randomized, controlled clinical trial was approved by the ethical review committee of Sichuan Provincial People's Hospital, Chengdu, China. Written informed consent was obtained from the parents or legal guardians of all children who agreed to participate. This study was conducted in full accordance with the World Medical Association Declaration of Helsinki.

### 2.1 Sample

The sample size was calculated using G*power 3.1 statistical software (G*power, Germany) for *t*‐tests with a one‐to‐one allocation ratio, assuming normality, for a two‐sided test. With α equals .05, based on a two‐sided test, a power of 95% and effect size as 58%, a total of 128 participants would be needed. We conducted our study from October 2019 to January 2020 in Sichuan Provincial People's Hospital, Chengdu, China.

### 2.2 Participants

For this study, 128 children were considered eligible. Children in the age group of 4–9 years, in good systemic health, without visual or auditory impairment, and requiring an extraction of primary teeth under local anesthesia were included. Children presenting with acute pain and requiring emergency dental treatment, suffering from any illness requiring special medical care, or uncooperative to finish the evaluation were excluded from the trial.

### 2.3 VR immersion system

**FIGURE 1:**
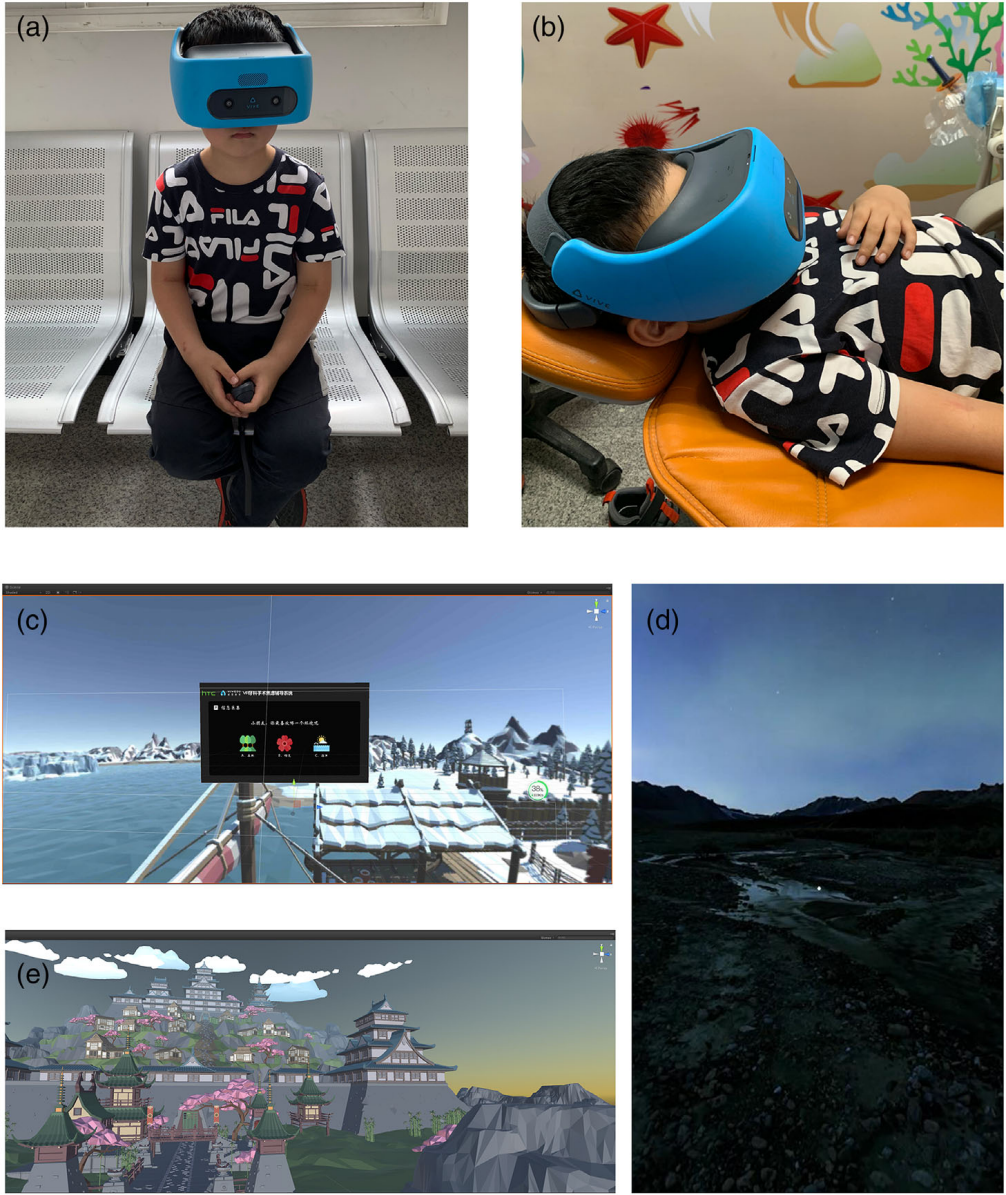
VR (virtual reality) system for children and screenshots of the virtual scenes. (a) It consists of a wireless head‐mounted helmet with noise‐reducing headphones and a controller. (b) Application of VR system during dental operation. (c) VR questionnaire before treatment. (d) Sakura scene for tell‐show‐do. (e) One relax scene during dental treatment

In our study, we used a VR system from HTC, which consisted of a wireless head‐mounted helmet with noise‐reducing headphones and a controller (Figure 1a,b). The host and base station are integrated with the head‐mounted helmet.

For this study, a medical software used VR system was customized (Vividu Chengdu, China). At first, a set of VR scene was used before dental treatment for children to be familiar with the equipment. A questionnaire investigating children's preferences was finished in the form of VR, which can help children choose immersion VR scene used in dental treatment (Figure 1c). The second set of VR scene was used before the dental treatment. Three different VR scenes (sea, forest, and sakura) were offered for children (Figure 1d). A tell–show–do interaction content was presented in the scene they chose. The Third set of VR scene was used during the treatment, that is a magic virtual world, which simulates scenes and sounds of nature (Figure 1e).

### 2.4 Intervention

The dental anxiety of the participants was measured using a modified Child Fear Survey Schedule‐Dental Subscale (CFSS‐DS) before dental examination by two trained dental assistants (Arapostathis et al., 2008). One assistant is responsible for explaining each item on the scale. If the child is too young, the parent can answer for him/her. The other assistant is responsible for recording and verifying. The modified CFSS‐DS questionnaire consists of 17 items and a 5‐point pictorial scale. The faces are scored 1−5, with 1 assigned to the most positive face and 5 assigned to the most negative face. The total score was 17–85, and the median was used to determine the level of anxiety in children. The higher the score was, the higher the degree of dental anxiety.

Then children were assigned by a single trained disinterested investigator to one of the groups (VR group or controlled group) by simple randomization (flipping of a coin) per patient (Suresh, 2011). During treatment, traditional behavior guidance procedures such as communication, tell–show–do, and reinforcement were used to help children adapt to the dental environment for the control group. In the VR group, we first applied a customized VR questionnaire to help children use VR system proficiently and choose their favorite virtual scene. Then, children in the VR group could get tell–show–do in their favorite virtual scene during treatment.

**FIGURE 2:**
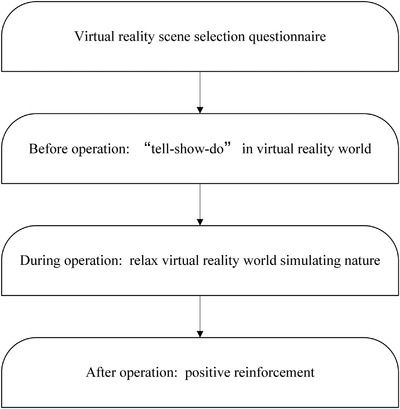
The flow chart for application of virtual reality during different operation stages

Topical anesthetic gel (Compound Lidocaine Cream, Tongfang, Beijing, China) was applied to the injection site by a sterile cotton applicator tip after drying with cotton gauze. The gel was rubbed on to the mucosa under moderate pressure for 30 se. After 3 min, the excess topical anesthetic was removed using a sterile cotton gauze. Then, the local anesthetic was administered by the buccal infiltration technique. One milliliter of 2% lidocaine was injected (lidocaine hydrochloride injection, Hualu, Shandong, China). We used the standard method of buccal infiltration technique described by Sridhar et al. (2019). Then, about 5 min later, the dentist separated the gum and extracted the primary teeth. During the procedure, the children in control group were given a standard set of verbal instructions regarding the injection with appropriate euphemisms, verbal reinforcement, and other traditional appropriate behavior management tools, while the VR group wore the helmet and interacted with the VR (Figure 2). All operations were completed within 20 minutes.

Wong‐Baker FACES Pain Scale was used for evaluation of the pain experienced by the children (Behavior Guidance for the Pediatric Dental Patient, 2011) just after the operation. The child was asked to choose the face that best describes own pain and record the appropriate number. At the same time, the dentist performing the operation assessed the behavior of children during treatment using Houpt scale (Houpt et al., 1985; Malhotra et al., 2016). The dental anxiety of the participants was measured using the CFSS‐DS after primary teeth extraction by the same two dental assistants. Then simulator sickness questionnaire (SSQ) was used to evaluate the incidence and severity of visually induced motion sickness in children after treatment (Prabhakar et al., 2007).

### 2.5 Statistical analysis

All data were analyzed using Statistical Package for Social Science (SPSS), version 16 (SPSS INC, Chicago IL, USA). The level of significance was set at 5% (*p* < .05). The quantitative data were described as (X ± s). The differences between the groups were compared using the Student's t test.

## 3 RESULTS

**FIGURE 3:**
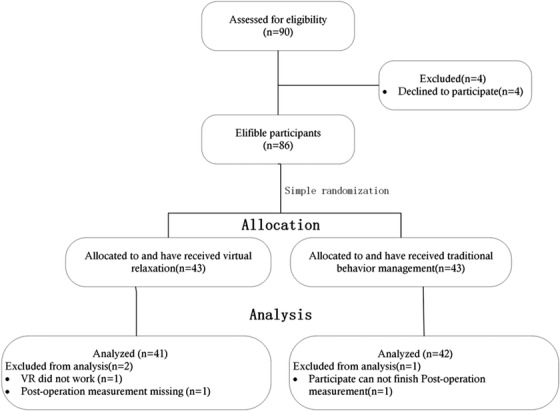
The flow chart of the randomized clinical study

The recruitment, randomization, allocation, and primary teeth extraction to the children belonging to the VR group and the control group are represented in the flow chart (Figure 3). A total of 128 children, which included 68 boys and 60 girls, were randomly divided into the VR group (*n* = 60) and the control group (*n* = 64). The age of children varied from 4.3 to 8.8, and the average age was 6.3± 3.5. Both the groups were comparable in terms of age (χ^2^ = 8.93, *p* = .063) and gender (χ^2^ = 0.25, *p *= .614) distribution, with no statistically significant difference between the groups.

We found that there is no significant difference in CFSS‐DS index between VR group and the control group before treatment (*p *= .612). But before and after the operation, there is a significant difference in the decrease of CFSS‐DS index in the VR group (*p *= .02), while there is no significant difference in the control group (*p *= .063), which suggests that the use of VR technology has a certain effect on reducing anxiety in children (Table 1).

**TABLE 1:** Intergroup comparison of dental anxiety and intragroup comparison before and after treatment

	CFSS‐DS (before operation)	CFSS‐DS (after operation)	*p*
VR group	34.58±6.90	32.32±15.58	.020
Control group	36.76±16.13	35.16±14.81	.063
*p*	.612	.385	

Abbreviations: CFSS‐DS, child fear survey schedule‐dental subscale; VR, virtual reality.

The WBFPRS index in the RA group is much higher than that in the control group. And there is a significant difference (*p *= 0.015). The results showed that the subjective sensation of pain in the experimental group was significantly lower than that in the control group (Table 2).

**TABLE 2:** Intergroup comparison of dental anxiety and intragroup comparison before and after treatment

	Wong Baker scoring	Houpt scoring
VR group	3.47±0.76	4.93±1.74
Control group	5.56±1.13	3.82±1.69
*p*	.015	.35

Abbreviation: VR, virtual reality.

Two children in the RA group were unable to complete the treatment, while in the control group, four children were unable to complete the treatment. Though the index in the VR group is higher than that in the control group, there is no significant difference (*p *= .35, Table 2).

No significant visual motion sickness was observed in the experimental group and the control group. There is no significant difference in the index of simulated motion sickness between the VR group and the control group (*p *= .305, Table 3).

**TABLE 3:** Intergroup comparison of incidence and severity of visually induced

	SSQ scoring	*p*
VR group	1.56±0.56	.305
Control group	1.24±0.51	

Abbreviations: SSQ, simulator sickness questionnaire; VR, virtual reality.

## 4 DISCUSSION

Digital technology is widely applied in medical treatment. Recently, FDA approved Akili Interactive prescription‐level electronic game EndeavorRx (AKL – T01) ADHD treatment for 8 to 12 years old boy; this is the first medical electronic game, which opened up a way of examination and approval for the therapy (Kollins et al., 2020). It marked the video game application is gaining recognition in the medical field.

VR technology is a combination of various technologies, including simulated environment, perception, natural skills, and sensing equipment. It is a generated simulation environment by computers, in which users can completely immerse (Iannicelli et al., 2019). The controller interacts with the virtual environment through a tactile feedback function. VR has been used for many medical purposes, such as medical training (Ayoub & Pulijala, 2019; Prabhakar et al., 2007), recovery of individuals with autism spectrum disorder (Saadatzi et al., 2018), and reduce pain and distress in invasive operation (Dumoulin et al., 2019; Iannicelli et al., 2019; Shetty et al., 2019).

Though the number of studies about VR applications to reduce anxiety, pain, and behavior in dental treatment is small. Different age groups, varies dental performed treatment, different VR equipment, and environment were discussed (Custódio et al., 2020; López‐Valverde et al., 2020). Most of the studies chose to focus on immersion in the pediatric population due to the attractiveness of VR for children and the challenge of children's behavior management during treatment (López‐Valverde et al., 2020). Restorative treatment, pulp therapy, local anesthesia, and extraction procedure are the most common dental procedures. Eyeglasses or helmets were usually used in the studies due to the portability.

In our study, we used a wireless head‐mounted helmet within host and base station. There was no need for linking it with computers or smartphones. Moreover, the head‐mounted helmet is totally enclosed. It provided a relatively isolated immersion VR environment. That maybe the main reason for the different results about the effectiveness of VR on dental anxiety reduction with studies before (Custódio et al., 2020; López‐Valverde et al., 2020). But the relatively isolated immersion VR environment may cause children's distress. It is necessary to help children adapt to it. Our research is the first to report the customized VR scene according to experimental requirements. It is interesting to find things that attract children for distraction. Customized VR scene is closer to the certain treatment children may face. Compared with usual video, the customized software can be more attractive, which maximizes the advantages of VR. Moreover, children can get more closed information about the treatment by an acceptable way and get positive reinforcement in a digital form.

Compared with traditional methods to reduce dental anxiety and pain, VR seems more attractive for children. And the content in VR is rich and variable according to requirement. It can distract children from dental treatment effectively, since they become more engaged in whimsical thinking and are fascinated by imaginative play (Attar & Baghdadi, 2015). Though VR technology has developed rapidly, there are still many shortcomings. One of the best known is cybersickness. VR may cause users to experience dizziness, vomiting, headaches, stomach upset, and nausea, a condition known as cybersickness (Weech et al., 2019, 2020). The main reason is that the motor information received by the vestibular system and the visual system does not match, and the conflict occurs when the brain tries to integrate the mismatched information (Koslucher et al., 2016; Palmisano et al., 2018). In our research, we controlled all operations within 20 min and examined the incidence and severity of cybersickness by the SSQ. The results show that no visual‐induced motion sickness was found both in the experimental group and the control group, which suggests that during the treatment period, children using the VR technology will not experience obvious vision‐induced motion sickness.

The pain experienced by the children was measured by Wong‐Baker FACES Pain Scale. The perception of pain is not a purely physical problem. It is influenced by social and psychological factors (Chapman & Nakamura, 1999; Garcia‐Larrea & Bastuji, 2018). The perception of pain includes a sensory discriminant component and a motivational affective component, both of which can be influenced by a variety of emotions such as fear of pain, anxiety, and depression (Thompson et al., 2016). In our study, the result shows that children's perception of pain in the RA group was significantly lower than the control group. We guess the use of RA helmets distracted the children by making the children unable to see the local acupuncture and tooth extraction. Niharika et al. (2018) also found that VR technology headsets can significantly reduce pain when children are treated with acupuncture and other operations. Other studies also show that VR technology is an effective method to reduce pain during children's treatment (Chad et al., 2018; Kennedy et al., 1993). In some operations, which may cause fear in children, such as “local anesthesia” and “extraction of baby teeth,” the application of VR headset with visual closure can help.

The dental anxiety was measured by using a modified CFSS‐DS. CFSS‐DS is a widely used international scale to measure children's anxiety index, which covers all aspects of children's anxiety in dental treatment (Arapostathis et al., 2008). The results suggested that the use of VR technology in the treatment process could effectively alleviate children's anxiety. The results are consistent with Niharika et al. (2018) and Laht et al. (2020). Although VR can only relieve anxiety to a certain extent, it cannot pacify patients with severe dental anxiety completely. For such children, we should combine with other effective behavioral management methods.

The application of VR technology in the extraction of children's primary teeth under local anesthesia can effectively reduce children's anxiety and pain feelings without cybersickness. But the use of VR is not adequate to help children with severe anxiety complete their treatment. There were also some limits in our study. Due to the use of helmets, there were no possibility to use the blind when we designed the trial. We investigated VR's effect on acute pain. More studies are needed to better understand the effect both on acute and chronic pain. With the rapid development and improvement of 5G technology, we expect more forms of VR technology, and personalized VR environment can be applied in clinical practice and promote the child's positive attitude toward oral health care.

## 5 CONCLUSIONS

The use of VR helmets in local anesthesia and primary teeth extraction can significantly reduce dental anxiety and pain perception in children without occurrence of simulator sickness. But it is not adequate to help children with severe anxiety complete their treatment.

## AUTHOR CONTRIBUTIONS

Du Qin and Ma Xinru are co‐first authors, and they contributed equally to this work. Du Qin obtained funding. Du Qin, Ma Xinru, and Liu Xianghong designed the study. Du Qin, Ma Xinru, Zhou Shiyu, and Luo Chunmei collected the data. Fei Wei, Ma Xinru, Wang Shasha, and Tian Kun were involved in data cleaning, verification, and analysis. Liu Xianghong drafted the manuscript. All authors have read and approved the final version of the manuscript. All authors revised the manuscript for important content and approved the final version.

## CONFLICT OF INTEREST

The authors declare no conflict of interest.

### PEER REVIEW

The peer review history for this article is available at https://publons.com/publon/10.1002/brb3.2600

## Data Availability

The data generated and analyzed in this study are available from the corresponding author upon reasonable request.
